# TRACE (Routine posTsuRgical Anesthesia visit to improve patient outComE): a prospective, multicenter, stepped-wedge, cluster-randomized interventional study

**DOI:** 10.1186/s13063-018-2952-5

**Published:** 2018-10-26

**Authors:** Valérie M. Smit-Fun, Dianne de Korte-de Boer, Linda M. Posthuma, Annick Stolze, Carmen D. Dirksen, Markus W. Hollmann, Wolfgang F. Buhre, Christa Boer

**Affiliations:** 1 Department of Anaesthesiology & Pain Medicine, Maastricht University Medical Centre +, P. Debeyelaan 25, 6229 HX Maastricht, The Netherlands; 2 Department of Anaesthesiology, Academic Medical Centre Amsterdam, Meibergdreef 9 H1Z-132, 1105 AZ Amsterdam, The Netherlands; 3 Department of Anesthesiology, VU University Medical Centre, De Boelelaan 1117, 1081 HV Amsterdam, The Netherlands; 4 Department of Clinical Epidemiology and Medical Technology, Maastricht University Medical Centre+, Maastricht, The Netherlands; 5 Care and Public Health Research Institute (CAPHRI), Maastricht University, Maastricht, The Netherlands

**Keywords:** Anesthesiology, Postsurgical complications, Failure-to-rescue, In-hospital mortality, Stepped-wedge cluster randomized trial

## Abstract

**Background:**

Perioperative complications occur in 30–40% of non-cardiac surgical patients and are the leading cause of early postoperative morbidity and mortality. Regular visits by trained health professionals may decrease the incidence of complications and mortality through earlier detection and adequate treatment of complications. Until now, no studies have been performed on the impact of routine postsurgical anesthesia visits on the incidence of postoperative complications and mortality.

**Methods:**

TRACE is a prospective, multicenter, stepped-wedge cluster randomized interventional study in academic and peripheral hospitals in the Netherlands. All hospitals start simultaneously with a control phase in which standard care is provided. Sequentially, in a randomized order, hospitals cross over to the intervention phase in which patients at risk are routinely followed up by an anesthesia professional at postoperative days 1 and 3, aiming to detect and prevent or treat postoperative complications. We aim to include 5600 adult patients who are at high risk of developing complications. The primary outcome variable is 30-day postoperative mortality. Secondary outcomes include incidence of postoperative complications and postoperative quality of life up to one year following surgery.

Statistical analyses will be performed to compare the control and intervention cohorts with multilevel linear and logistic regression models, adjusted for temporal trends and for clusters (hospitals). The time horizon of the economic (cost-effectiveness) evaluation will be 30 days and one year following surgery.

**Discussion:**

TRACE is the first to study the effects of a routine postoperative visit by an anesthesia healthcare professional on mortality and cost-effectiveness of surgical patients. If the intervention proves to be beneficial for the patient and cost-effective, the stepped-wedge design ensures direct implementation in the participating hospitals.

**Trial registration:**

Nederlands Trial Register/Netherlands Trial Registration, NTR5506. Registered on 02 December 2015.

## Background

Perioperative complications occur in approximately 30–40% of non-cardiac surgical patients [1] and are the leading cause of early postoperative morbidity and mortality [2]. Moreover, the occurrence of early complications seems to be associated with long-term mortality that persists for years after surgery [3]. Several studies have demonstrated that surgical complications increase the overall cost of care [4–6]. Therefore, avoiding or even early treatment of complications is valuable in terms of cost-effectiveness [5, 7].

Although there is little variation in the occurrence of complications among hospitals, the resulting in-hospital mortality related to perioperative complications varies considerably [1]. In 1992, Silber et al. created the term failure-to-rescue to describe the percentage of deaths in relation to complications per surgical procedure [8]. Depending on the type of surgery, the failure-to-rescue rate varied widely across hospitals [9, 10]. Most likely, some organizations are able to cover the needs of patients with complications better than others [1, 8, 11, 12]. A more recent trial from the ISOS group observed a failure-to-rescue rate in the range of 1.7–3.6% in a mixed cohort of more than 44,000 patients in 27 countries [13].

Factors contributing to the development of failure-to-rescue have been studied intensely over the past 15 years; however, most of the data come from national or institutional databases and only little is known about failure-to-rescue rates in Europe [13]. It has been hypothesized that regular visits by highly educated and trained health professionals such as intensive care physicians, surgeons, or anesthesiologists can contribute to a decreased incidence of complications by earlier detection and adequate treatment.

Until now, no studies have been performed on the impact of routine postsurgical anesthesia visits on the incidence of postoperative complications. Therefore, we designed a nation-wide study in which patients at risk are routinely followed up by an anesthesia professional at postoperative days 1 and 3, aiming to detect and prevent or treat postoperative complications. Based on the available literature, we hypothesized that routine postsurgical anesthesia visits decrease the incidence of failure-to-rescue and reduce postoperative 30-day mortality by 50%.

### Research questions

1. What is the incidence of postoperative complications in this study cohort?
2. Is there an impact of regular anesthesia visits on postoperative days 1 and 3 in reducing postoperative complications, 30-day mortality, and length of stay?
3. What is the impact of the routine anesthesia visits on the time to implementation of rescue therapy, the utilization of high-care and intensive care facilities after complications, and the length of stay in these units?
4. What is the impact of routine anesthesia visits on the quality of recovery 30 days after surgery and on the quality of life one year after surgery?
5. Is the implementation of routine anesthesia visits after surgery cost-effective compared to standard postoperative care?

## Methods/Design

### Study design

The TRACE study is a multicenter prospective interventional study with a stepped-wedge design, aiming to recruit 5600 patients. In the beginning of the study all participating medical centers will provide perioperative care using the standard approach. All patients included during the standard approach period will be enrolled in the control group. Stepwise and at equal time periods, interventional postoperative anesthesia care will be implemented in the participating centers. The order in which the centers start with the intervention is randomized. The additional anesthesia care will consist of anesthesia visits on postoperative days 1 and 3 to follow up patient care and enhance rescue therapy when needed. By the end of the study, all participating centers will have implemented the intervention.

### Study schedule and randomization

**Fig. 1:**
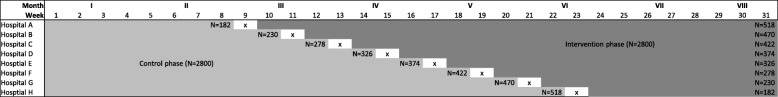
Stepped-wedge schedule of the TRACE Study. Hospitals sequentially cross over from control phase to intervention phase. Transistion periods between phases are depicted by ‘x’

Figure 1 shows the planned study schedule. Total recruitment time covers 31 weeks. All hospitals start in week 1 in the control phase and after week 8 sequentially cross over to the intervention phase. Between the two phases there is a one-week transition period (depicted with an *x*). In week 24, all hospitals will have started the intervention phase.

In total, 2800 patients will be recruited in the control phase and 2800 in the intervention phase. Each hospital will recruit 700 patients. The first hospitals to cross over (A–D) will recruit more patients in the intervention phase than in the control phase and the last hospitals (E–H) will recruit more patients in the control phase than in the intervention phase.

Randomization for the order in which hospitals cross over from the control phase to the intervention phase was performed before the first patient was recruited. The names of the eight hospitals were ordered randomly using envelope drawing. This randomized order was translated into the schedule presented in Fig. 1.

### Inclusion and exclusion criteria

#### Inclusion criteria

Patients undergoing elective surgery with an indication for postoperative hospital stay can be included in the study if they meet at least one of the following criteria:

- Aged ≥ 60 years;
- Aged ≥ 45 years with a revised cardiac risk index (rCRI) > 2;
- Aged ≥ 18 years with an indication for postoperative invasive pain therapy;
- Aged ≥ 18 years with a postoperative surgical APGAR-score (sAPGAR) < 5.

#### Exclusion criteria

Patients undergoing cardiac surgery and patients with an indication for a postoperative stay in the intensive care unit.

### Recruitment and consent

Patients will be recruited by a member of study team (anesthesiologist or research assistant) preoperatively, either during the preoperative screening or directly after hospital admission. Patients receive a written patient information letter and are additionally orally informed about the study aims and the study phase they will enter (control or intervention). If they agree to participate, they will be asked to sign informed consent.

### Intervention and control condition

During the intervention phase, all participants will receive a postoperative visit from the anesthesiologist at postoperative days 1 and 3. The postoperative visit is standardized and based on the Modified Early Warning Score (MEWS). The MEWS includes the following measurements: respiratory rate; heart rate and rhythm; systemic oxygen saturation; systolic blood pressure; body temperature; level of consciousness; and urine output. Other measurements include: visual analogue score to assess pain during rest and movement; nausea/vomiting; defecation; and mobilization. In case of a higher MEWS score and/or other concerns, the anesthesia health professional will advise the treating physician on further diagnosis, treatment, and follow-up of the patient.

During the control phase, participants will receive standard care, without a standardized visit from the anesthesiologist. The treating physician may ask the anesthesiologist in consultation when deemed necessary. Standard of care may slightly differ across the different participating hospitals.

### Participating centers

The study will be performed in eight Dutch hospitals, representing general hospitals, tertiary referral hospitals, and academic centers. All participating hospitals received approval from the Board of Directors to participate in the TRACE study.

### Data collection

Patient and clinical data will be collected at several time-points during the study period: at inclusion (baseline); intraoperatively; postoperatively at days 1 and 3; during hospital stay; from hospital discharge until 30 days; and until 12 months. Data records will be coded and transcribed by local investigators into an Internet-based electronic case record form.

Data to be collected from patient record files include patient baseline characteristics, data on surgery and anesthesia, intraoperative adverse events, the postoperative clinical course, postoperative in-hospital adverse events, and post-discharge events measured at 30 days and at 12 months after the date of surgery. During the intervention period, data collection additionally includes anesthesia interventions on postoperative days 1 and 3.

**Fig. 2:**
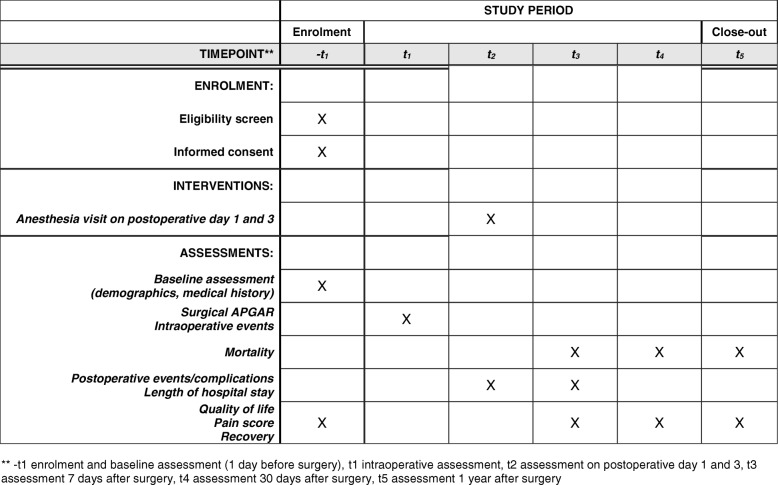
TRACE study schedule of enrolment, interventions, and assessments

Data will also be collected from patient questionnaires, completed at inclusion, seven days, 30 days, and 12 months postoperatively. The questionnaires include questions on quality of life (EuroQol Dutch EQ-5D-5 L), pain scores (numeric rating scale, NRS), functional recovery (Functional Recovery Index), and expected/perceived recovery (Global Surgery Recovery index). See Fig. 2 for an overview of enrolment, intervention, and assessments.

### Outcome measures

The primary outcome measure will be the mortality at 30 days after surgery. Secondary outcome measures will be incidence of postoperative complications (in eight domains—infectious, cardiac/transfusion, pulmonary, venous thromboembolic, renal, neurological, surgical, and other—in line with Meguid et al. [14]), time to implement rescue therapy, length of stay in hospital, length of stay in a high-care unit, quality of recovery 30 days after surgery, and postoperative quality of life up to one year following surgery.

### Sample size calculation

The calculation is based on the primary outcome parameter 30-day mortality. In an earlier Dutch study, postoperative all-cause death was 1.85% [15]. Recently, the EUSOS study observed a mortality rate of approximately 2% [16], which is in line with previous results.

We considered a difference of 50% between monitored and non-monitored patients to be clinically relevant. In order to detect a reduction from 2% to 1% in postoperative 30-day mortality, with an alpha of 0.05 and a power of 80%, and a 1:1 ratio between control and intervention group, a total of 4638 patients have to be included in a parallel randomized controlled trial (RCT) design. To compensate for inter-institutional variation, a possible time-effect inherent to the stepped-wedge design, and drop-out, we increased this sample size with 20%. The relatively uncommon approach of applying a 20% inflation factor was discussed extensively during the grant application and ethical approval processes and was finally approved by both the Netherlands Organisation for Health Research and Development and the Medical Ethical Commitee.

Thus, we estimate a requirement of 5600 patients. With eight participating hospitals, this translates into 700 patients per hospital. Figure 1 illustrates how these patients will be divided across hospitals and between the control and intervention groups.

### Statistical analysis

Primary and secondary outcome measures (proportions with 95% confidence intervals or means with standard deviations) will be calculated and presented in tables. A patient flow chart will be presented to account for inclusions per hospital and drop-out.

For the primary outcome and binary secondary outcomes, multi-level mixed effect logistic regression with random time by cluster interaction will be used to compare between the cohort that has not and the cohort that has received the intervention according to the stepped-wedge schedule. Time effects will be modelled according to appropriate methods, e.g. using spline functions. For continuous secondary outcomes, multi-level mixed effect linear regression will be used and for countable secondary outcomes, multi-level mixed effect Poisson regression models will be used. Analyses will be performed using SPSS and R.

On cluster level, per-protocol analyses will be performed (i.e. if the intervention phase starts later than expected in a certain hospital, patients will be allocated to the control group until the hospital has actually crossed over). Once the intervention phase has started, individuals within that cluster will be analyzed according to the intention-to-treat principle. Adjustments will be made for temporal trends and for clusters within hospitals [17]. An economic evaluation from a hospital perspective will be performed for postoperative anesthesia follow-up of high risk surgical patients compared to standard postoperative care. The time horizon of the economic evaluation will be 30 days and one year following surgery. For reasons of efficiency, the analysis after one year will be performed in a subset of study participants. A cost-effectiveness analysis will be performed, based on the incremental costs per postsurgical visit. Standard sensitivity analysis will be performed to test whether results are robust for changing certain parameters, e.g. cost-prices. Confidence intervals surrounding the mean differential costs will be calculated by the bootstrap method. The latter will also be used to quantify the uncertainty surrounding the incremental cost-effectiveness ratio [18, 19]. Additionally, a budget impact analysis will be performed to assess the affordability of a routine postoperative visit by an anesthesia healthcare professional by relating the increase in cost due to investment in anesthesia professionals to the cost-savings due to the expected reduction in complications and hence length of hospitalization and mortality.

### Trial status

At the time of initial manuscript submission, recruitment had started (November 2016) but has not been completed. We expect recruitment to be completed in September 2018. The current protocol version is version 5 (3 January 2018).

## Discussion

To our knowledge, the TRACE study is the first to study the effects of a routine postoperative visit by an anesthesia healthcare professional on the mortality and cost-effectiveness of surgical patients.

We have chosen for a stepped-wedge design because randomization on a patient level or cluster level (e.g. a hospital ward) was not feasible. It is logistically very challenging to have two parallel care paths within a hospital, for example in a RCT, where there is a chance of information transfer because the same healthcare professionals have to apply two different forms of postoperative care. Cluster randomization is only possible with certain heterogeneity within wards; however, this is not the case because patients with different conditions are not randomly distributed over hospital wards. An important advantage of the stepped-wedge design is that the design ensures implementation of the intervention in all centers. Thus, no hospitals will be withheld from the new approach and the implementation phase follows naturally after the research phase.

The TRACE study introduces a change in postoperative patient care by the anesthesiologist that was set up in collaboration with surgical specialists. The knowledge and skills of the anesthesiologist as perioperative physician are believed to benefit the patient. In practice, it may be difficult to separate beneficial effects by the anesthetist from enhanced care by the surgeon and ward physician. Therefore, with this study we aim to gain insight into whether the introduction of postoperative monitoring by the anesthesiologist leads to more monitoring/consults by the ward physician or nurses. This could be a beneficial side effect of the intervention to the patient.
